# The simultaneous occurrence of multiple myeloma and JAK2 positive myeloproliferative neoplasms - Report on two cases

**Published:** 2015

**Authors:** S Badelita, C Dobrea, A Colita, M Dogaru, M Dragomir, C Jardan, D Coriu

**Affiliations:** * Centre of Hematology and Bone Marrow Transplant, “Fundeni” Clinical Institute, Bucharest, Romania; ** “Carol Davila” University of Medicine and Pharmacy, Bucharest, Romania

**Keywords:** multiple myeloma, primary myelofibrosis, essential thrombocythemia, JAK2

## Abstract

Multiple myeloma and JAK2 positive chronic myeloproliferative neoplasms are hematologic malignancies with a completely different cellular origin. Two cases of simultaneous occurrence of multiple myeloma, one with primary myelofibrosis and another one with essential thrombocythemia are reported in this article. In such cases, an accurate diagnosis requires a molecular testing, including gene sequencing and differential diagnosis of pancytosis associated with splenic amyloidosis. In general, in such cases, of two coexisting malignant hematologic diseases, the treatment of the most aggressive one is recommended. For our two cases, it was decided to start a Velcade based therapy. The main concern was the medullar toxicity, especially when a multiple myeloma was associated with a primary myelofibrosis.

**Abbreviations**:JAK2 = Janus kinase 2 gene, PMF = primary myelofibrosis, MPNs = myeloproliferative neoplasms, ET = essential thrombocythemia, PV = polycythemia vera, MM = multiple myeloma, WBC = white blood cells, Hb = haemoglobin, Ht = haematocrit, Plt = platelets, BMB = bone marrow biopsy, CBC = blood cell count, CT = computerized tomography, LAP = leukocyte alkaline phosphatase, MGUS = monoclonal gammopathy of undetermined significance.

## Introduction

Multiple myeloma (MM) is a chronic malignant lymphoproliferation originating in B cell post - germinal center which has undergone somatic mutations and which has the ability to differentiate into plasma cells [**1**].

Philadelphia chromosome negative myeloproliferative neoplasms (MPNs) are a heterogeneous group of chronic diseases characterized by the cell proliferation of one or several hematopoietic lines. They are clonal diseases originating in a pluripotent myeloid hematopoietic stem cell that can differentiate between erythroid progenitors, granulocytic progenitors and the megakaryocytic progenitors [**2**]. This group of diseases includes primary myelofibrosis (PMF), essential thrombocythemia (ET) and polycythemia vera (PV) [**3**]. Nearly all the patients (95%) with PV have the V617F somatic mutation present in the Janus kinase 2 gene (JAK2). This mutation is also found in 65% of the patients with PMF, respectively 55% of the patients with TE.

The cases of two patients with multiple myeloma associated with primary myelofibrosis and, respectively, essential thrombocythemia, are presented in this article.

## Case Report

**Case 1**

A 65-year-old patient with multiple comorbidities (previous surgery for aortic stenosis - metal prosthesis, peripheral vascular disease with angioplasty with stent in the left common iliac artery, with episodes of paroxysmal atrial fibrillation, ischemic nephropathy with renal artery stenosis, and chronic kidney disease – 2nd degree), was admitted for investigation in the Hematology Centre of “Fundeni” Clinical Institute, Bucharest, for hepatosplenomegaly (liver diameter of 19.5 cm, homogeneous splenomegaly - bipolar diameter 16.7 cm), incidentally discovered on a routine ultrasound scan.

Blood cell count (CBC) showed leukocytosis with immature granulocytes and erythroblasts on peripheral smear, with macrocytes, anisocytosis, poikilocytosis, and red blood cells inclusions and teardrop-shaped RBC. (WBC = 10,540/ mmc - Metamyelocytes 1, Bands 4, Neutrophils 72, Eosinophils 2, Basophils 2, Lymphocytes 12, Monocytes 7; Hb = 12 g/ dl, Hct = 35.9%, Plt = 407,000/ mmc).

Renal tests revealed chronic kidney disease stage III B (creatinine = 2.55 mg/ dl, creatinine Clearance = 42.08 ml/ min.), and viral markers (HBsAg, HCV Ab, HIV) were negative.

**Fig. 1:**
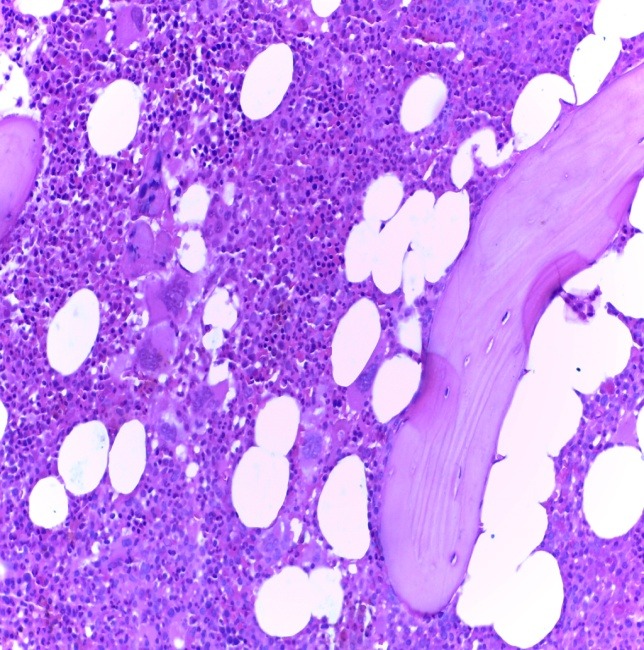
BMB Hypercellular marrow, megakaryo-granulocytes proliferation, clustered polymorphous atypical megakaryocytes (HE stain, ob x20)

**Fig. 2:**
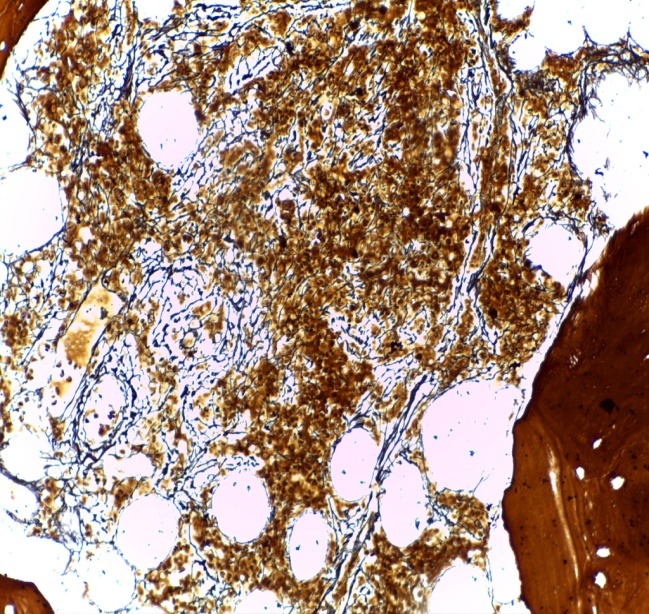
2 BMB – Grade 2 fibrosis (Gomori stain, ob x20)

Bone marrow biopsy (BMB) examination showed a hypercellular marrow (about 80% marrow cellularity), with pronounced proliferation of megakaryo- and granulopoiesis: very frequently polymorphous megakaryocytes, with an atypical morphology (from giant megakaryocytes with cloud-like, bulbous nuclei to small dwarf megakaryocytes with hyperchromatic nuclei); megakaryocytes were densely clustered perivascularly and paratrabecularly; sinusoids were proliferated, without intravascular hematopoiesis (**Fig. 1**). Gomori silver stain for fibrosis showed grade 2 myelofibrosis (**Fig. 2**). The pathological diagnosis was PMF, fibrotic hypercellular stage.

**Fig. 3:**
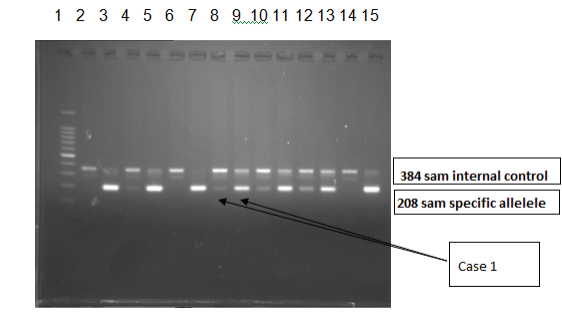
ARMS-PCR method used for detection of the V617F mutation in JAK 2 gene positive for Case 1. 1st lane – Molecular weight marker (of 100smp in 100 smp); 2nd 9th lanes – Pacient samples 2 locus for each pacient; 10th-11th lanes – Positive control; 12th-13th lanes – Negative control; 14th-15th lanes – Blank. The first lane of each patient is mutant allele (JAK2 V617F) and the 2nd is the normal allele (wildtype JAK2)

**Fig. 4:**
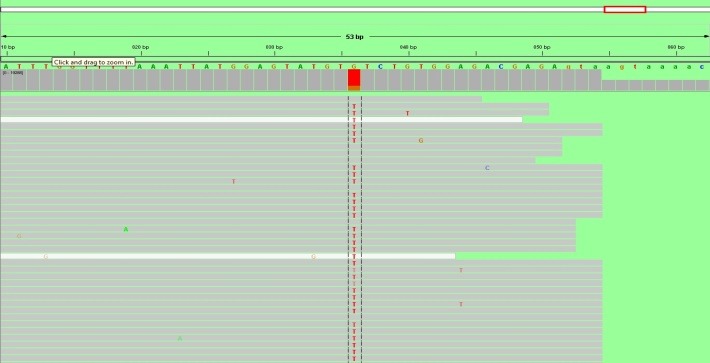
Aligned sequences showing mutation in JAK2 exon 14 - c1849 G>T (V617F). Prevalence of this mutation in this patient was 72%. Sequencing was performed on Miseq Illumina. For JAK2 exon 12, 13 and 14 sequencing, specific primers, which amplify exon 12, 13 and 14, were used. After amplification, amplicons were purified by using Agencourt Ampure XP magnetic beads following the manufacturer's protocol. Purified amplicons were indexed by using indexes provided by Illumina and resulting fragments sequenced on Miseq sequencer from Illumina, after another round of purification by using Agencourt Ampure XP. Sequencing was performed by using MIseq kit Nano and 2x250 sequencing protocol. Resulting sequences were aligned by using LaserGene 11.2 software and visualized and analyzed by using IGV version 2.3.40. An average coverage of 10000x was obtained for all exons.

In the context of histopathological suspicion for chronic myeloproliferative neoplasia, investigation was supplemented with Leukocyte Alkaline Phosphatase (LAP) = 145 (n: 10-100) and molecular biological testing - analyzing the DNA sample by Amplification - Refractory Mutation System (ARMS) – PCR for V617F somatic mutation in JAK2 gene [**4**,**5**]. This mutation was identified in heterozygous form in the JAK2 gene (**Fig. 3**). This result was confirmed by JAK2 gene sequencing using next generation sequencing (**Fig. 4**).

The diagnosis of “primary myelofibrosis” was established and it was considered that the patient did not require a specific hematologic treatment; the patient should be monitored at every 3 months.

After 6 months, the patient presented thoracolumbar spine pain, right paravertebral muscle contraction, and weight loss. CT scans showed multiple osteolytic lesions in the ribs and the scanned thoracic lumbar spine, and paravertebral tumor with the involvement of the spinal canal at T10. As the patient had motor neurological lesions with incomplete neurological injury Frankel C - T9 level, and retention of urine rapidly installed, he was urgently admitted to the Clinic of Neurosurgery, where a T10 spinal tumor ablation was performed, together with a reconstruction with polyacrylic cement, and metal synthesis T8, T9, T11 on the right side.

Patient discharge: 4/ 5 predominantly proximal paraparesis, bilateral Babinski post spinal cord compression, Bilateral ataxic syndrome.

**Fig. 5:**
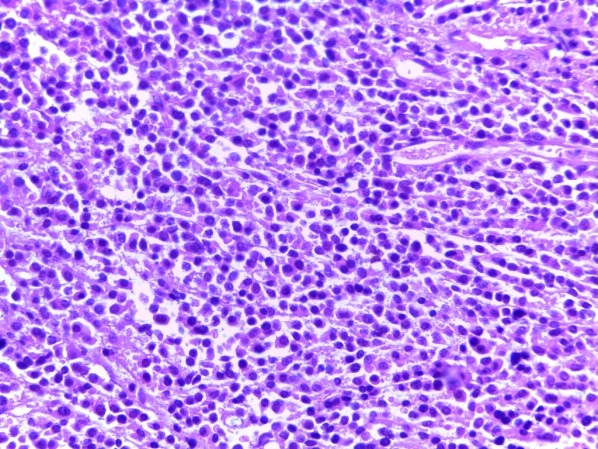
Vertebral tumor – Extramedular infiltration of plasma cell myeloma (HE stain, ob 40x)

Histopathology of tumor showed plasmacytic plasmacytoma/ extramedular infiltration of MM; Congo Red stain was negative (**Fig. 5**).

In evolution, blood cell counts showed normochromic normocytic anemia and left shift of leukocyte formula. St. III B chronic kidney disease remained present while the subnephrotic rank of proteinuria began to appear (Proteinuria = 0.71g/ 24h).

**Fig. 6:**
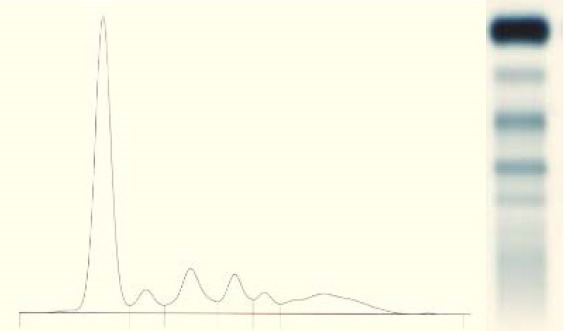
Serum protein electrophoresis, no monoclonal spike; normal values

Serum protein electrophoresis: Total proteins = 5.6 g/ dl; normal appearance; normal values of serum Immunoglobulins (**Fig. 6**).

Serum free light chain kappa = 22.8 mg/ l (N 3,3 – 19,4), serum free light chain lambda = 1840 mg/ l (N 5,71 - 26,3), Ratio free light chain kappa/ lambda = 0.0123 (N 0,26 – 1,65) [**10**].

**Fig. 7:**
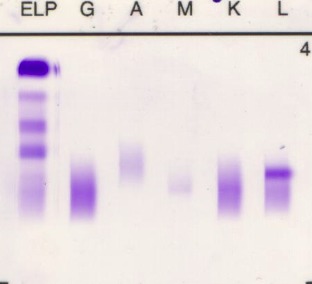
Immunofixation electrophoresis: positive for light lambda chain

Immunofixation electrophoresis: positive for light lambda chain (**Fig. 7**).

**Fig. 8:**
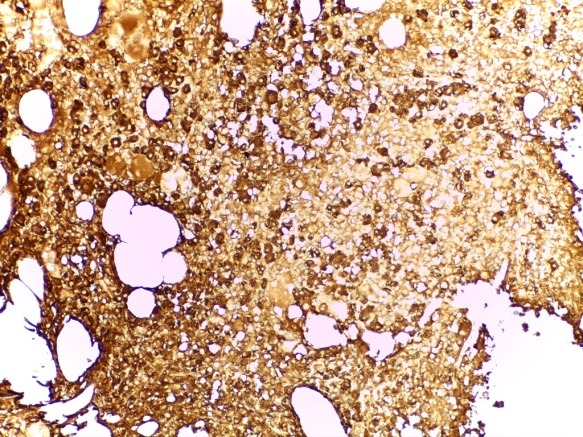
BMB IHC examinatin - interstitial lambda chain myelomatous infiltrate (IHC stain for lambda light chain, ob x20)

The immunohistochemical (IHC) reevaluation of the BMB with AE1/ AE3 pan cytokeratin and kappa and lambda light chains showed interstitial plasma cells clonal infiltration (approx. 20-22%) with lambda light chain restriction (**Fig. 8**); AE1/ AE3 was negative. The final histopathological diagnosis was PMF – hypercellular fibrotic stage, associated with plasmacytic MM with lambda light chain restriction. 

The diagnosis of “Lambda light chain Multiple Myeloma stage III B; T10 vertebral plasmacytoma; JAK2 positive Primary Myelofibrosis” was done and it was decided to urgently start the treatment of multiple myeloma due to the advanced stage of the disease (anemia and significant bone mass loss), opting for the monitoring of PMF, as, at that time, the patient did not present any notable cytopenia/ pancytosis.

Due to the multiple associated comorbidities and the patient’s fragile clinical status, we decided to initiate therapy according to VelDex (Velcade, Dexamethasone) protocol and bisphosphonates.

**Fig. 9:**
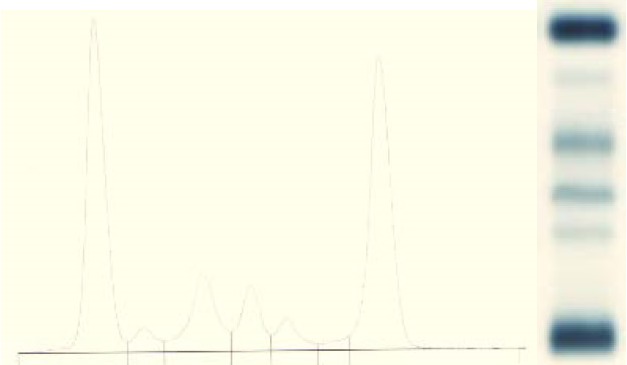
Serum protein electrophoresis: monoclonal IgG M- spike 6,08 g/ dl

**Case 2**

A 71-year-old female patient, known with invasive ductal breast carcinoma treated with mastectomy, chemotherapy and local radiotherapy (13 years before), has also presented thrombocytosis (Tr> 700,000/ mmc) for approximately 7 years, for which she received aspenter treatment.

Four years before, the patient was admitted in a Hematology Department to investigate that thrombocytosis; CBC showed thrombocytosis (Plt = 840,000/ mmc) with anisocytosis and macro platelets; normal results on clinical examination and imaging investigations (CT scan, bone scintigraphy and ultrasound). BMB examination showed a normal age-related marrow cellularity with mildly increased megakaryocytes: small loose clusters of large to giant megakaryocytes, with mature cytoplasm and multilobulated staghorn-like nuclei. The pathology of BMB was compatible with an ET. In addition, the investigations were supplemented with molecular biology tests - analyzing the DNA sample by Amplification - Refractory Mutation System (ARMS) – PCRfor V617F somatic mutation in JAK2 gene. This mutation was identified in a heterozygous form in the JAK2 gene. The diagnosis was “Essential thrombocythemia” and treatment with Anagrelide was started. During treatment, the patient presented mild anemia (Hb = 10.9 g/ dl) and normal value platelets. Anemia was interpreted as a side effect of Anagrelide.

The patient was admitted to the Hematology Centre of “Fundeni” Clinical Institute with mild skin pallor, ribs, spine and pelvis bone pain (started within the previous year), without organomegaly. The patient had not taken Anagrelid within the last three months.

CBC: WBC = 8400/ mmc (normal white blood count), Hb 9.3 g/ dl, Hct 29.4%, Plt = 243,000/ mmc, rouleaux distribution of erythrocytes, without Howell - Jolly bodies. Coagulation tests and platelet aggregation in normal limits. Normal LAP.

Serum protein electrophoresis: Total proteins = 10,1 g/ dl; Alb = 33.7%, Alfa1 = 3.2%, Alfa 2 = 12.4%, Beta 1 = 7.8%, Beta 2 = 4.2%, Gamma 1 = 1.6%, Gamma 2 = 37.1%, Monoclonal band in gamma globulin area = 3.7 g/ dl, Ig G = 2910 mg/ dl (Normal 700-1600 mg/ dL, Ig A < 35.4 mg/ dl (70 – 400 mg/ dL), Ig M = 37.2 mg/ dl (40 – 230 mg/ dL).

**Cryoglobulins present in deposit**

Immunofixation electrophoresis (including within cryoprecipitate): positive for heavy Ig G chain and light Kappa chain [**8**].

Serum free light chain kappa = 3.12 mg/ l (N 3,3 – 19,4), Serum free light chain lambda = 646 mg/ l (N 5,71 - 26,3), Ratio kappa/ lambda = 0.0048 (N 0,26 – 1,65).

The imaging tests performed were: skeletal radiography (which showed a score > 3 osteolytic lesions at skull level); Magnetic Resonance Imagining for the spine and pelvis, revealing diffuse bone structural changes affecting the lumbar vertebral floor and the pelvic bones, suggestive of a tumor substrate) and abdominal ultrasound (which did not reveal hepato-splenomegaly).

**Fig. 10:**
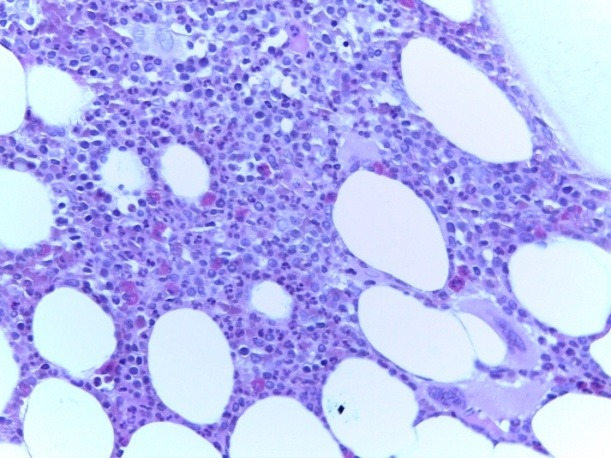
BMB Hypercellular marrow by diffuse plasma cells infiltrate; small clusters of giant megakaryocytes (HE stain, ob x40)

**Fig. 11:**
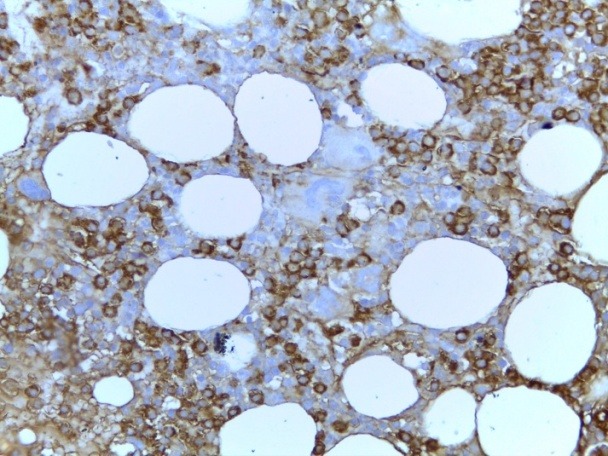
BMB Plasma cells infiltrate was clonal, with lambda light chain restriction (IHC stain for lambda light chain, ob 40x)

The bone marrow biopsy was repeated and this showed a hyper cellular marrow with diffuse atypical plasma cells infiltration (approx. 60%); small clusters of giant megakaryocytes still remaining (**Fig. 10**). IHC stain showed clonality of marrow plasma cell infiltrate, with lambda light chain restriction (**Fig. 11**). The pathological diagnosis was lambda light chain MM associated with ET.

An abdominal fat pad biopsy was negative for Congo red stain. No signs of amyloidosis were identified.

**Fig. 12:**
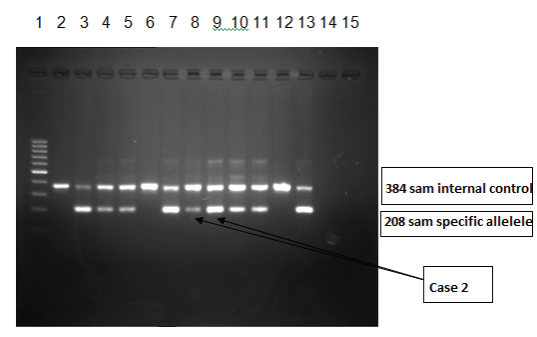
ARMS-PCR method for the detection of V617F mutation in JAK 2 gene low level positive for Case 2. 1st lane – Molecular weight marker (of 100 samples/ s in 100s); 2nd 9th lane – Pacient samples 2 lanes for each pacient; 10th - 11th lane – Positive control; 12th-13th lane – Negative control; 14th-15th – Blank. The first lane of each patient is mutant allele (JAK2 V617F) and the 2nd is the normal allele (wildtype JAK2)

**Fig. 13:**
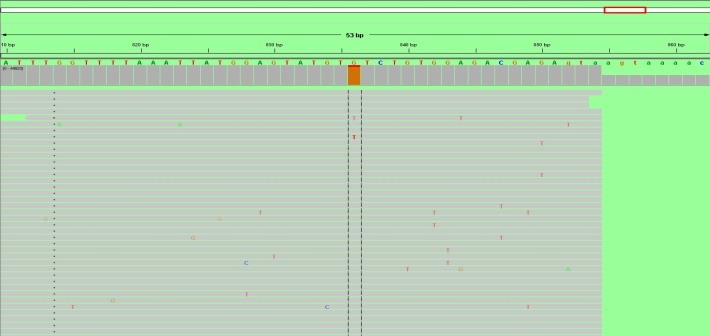
Aligned sequences showing mutation in JAK2 exon 14 - c1849 G>T (V617F). Prevalence of this mutation in this patient was 9%. Sequencing was performed on Miseq Illumina

The molecular biological tests were repeated, showing the V617F somatic mutation allele in the JAK2 gene low level positive (**Fig. 12**) in the DNA sample analyzed by ARMS – PCR. This result was confirmed by JAK2 gene sequencing by using next generation sequencing (**Fig. 13**).

Considering all the investigations performed, the diagnosis of “Multiple myeloma IgG lambda stage III A associated with type I cryoglobulinemia; JAK2 positive Essential Thrombocythemia” was established and it was considered necessary to start the treatment for multiple myeloma, due to the advanced stage of the disease and the presence of cryoglobulinemia, while there were no criteria for starting or continuing the treatment for ET.

We decided to initiate therapy according to the CyBorD (Velcade, Cyclophosphamide and dexamethasone) protocol and bisphosphonates.

Regarding the treatment of multiple myeloma for the two cases presented, it is to be noted that these were patients older than 65 years, who had multiple comorbidities and were not eligible for autologous bone marrow transplant consolidation; and, for this reason, they continued the treatment based on bortezomib regimen. Subsequently, the patient was monitored once at every 3 months (blood cell counts, kidney function, blood viscosity, 2 beta microglobulin, lactic dehydrogenase test, serum protein electrophoresis, Ig level, monitoring of serum free light chains assays), bone marrow aspiration and/ or imagistic evidence (Skeletal Radiography, Magnetic Resonance Imaging). The treatment with bisphosphonates was continued monthly for a period of 24 months.

In terms of chronic myeloproliferative syndrome, at every 3 months, blood cell count with differential white blood count and an abdominal ultrasound was performed.

## Discussions

The association of multiple myeloma with chronic myeloproliferative neoplasms is a rare occurrence. Malhotra [**6**] reported 15 patients who were diagnosed with MPN and plasma cell disorder such as multiple myeloma or monoclonal gammopathy of undetermined significance (MGUS). Most of these patients were diagnosed with MPN prior to or simultaneously with MM or MGUS.

From our perspective, the association of these two diseases raised a few issues that are very important in theory, but also clinically:

- The issue of the relationship between the two malignancies occurring in the same patient:

Is there a common hematopoietic stem cell for MM and MPN? This is an unsustainable hypothesis, since there is data demonstrating the cellular origin of MPN in a myeloid hematopoietic stem cell, while MM originates in the B lymphocyte, which suffered somatic mutations in the germinative center.

Regarding the issue of whether the therapy administered for a disease influenced the development of a second neoplastic disease - in the first case, this was out of the question, since the two diseases were diagnosed almost simultaneously. In the second case, there was room for discussion - the patient was known with a history of breast cancer treated with an intense chemo and radiation therapy.

- The diagnosis issue: 

In the first case of JAK2 positive primary myelofibrosis preceding with 6 months the diagnosis of multiple myeloma, the evolution was practically simultaneous. The advanced bone marrow fibrosis associated with multiple myeloma was found in 20.5% out of 44 patients with multiple myeloma and was a negative prognosis factor – with an average survival period of 11 months [**7**]. In our case, the initial diagnosis was primary myelofibrosis with all the specific criteria, including the molecular ones and the diagnosis of multiple myeloma was missed, even though when we reassessed the bone marrow biopsy, the signs of plasma cell monoclonal proliferation were found. In terms of histopathology, the presence of bone marrow fibrosis (that modifies the bone marrow tissue architecture) can delay the identification of small associated plasma cells infiltrates, requiring further investigations (e.g. immunohistochemistry) and a correlation with the clinical data [**9**,**12**]. On the other hand, a significant bone marrow plasma cell infiltration dislocates the normal hematopoiesis to a variable degree, making it difficult to pathologically sustain the diagnosis of associated myeloproliferative neoplasm and its differentiation from secondary megakaryocyte hyperplasia to a monoclonal gammopathy. Moreover, the difficulty of the multiple myeloma diagnosis, in this case, is enhanced by the fact that the clonal population of plasma cell secrete only free light chain (micromolecular multiple myeloma), the serum protein electrophoresis appearance is normal, and the final serological diagnosis can be established only by the free light chains assay or by the serum protein immunofixation.

In the second case, the diagnosis of ET was established four years before the development of the multiple myeloma criteria. In our experience, some cases of multiple myeloma and associated amyloidosis (AL amyloidosis) have leukocytosis, thrombocytosis and/ or erythrocytosis. Cases of primary amyloidosis were reported to start as pancytosis by amyloid related functional hyposplenism [**11**]. The peripheral blood smear examination and identification of Howell - Jolly bodies in the red cells are very important in all these cases.

- The therapy issue:

Generally, in such situations where two hematological malignancies coexist, we recommend the treatment of the most aggressive disease. In our case, it was necessary to start the Bortezomib - based therapy. Of course, the primary concern was the bone marrow toxicity, especially when a multiple myeloma was associated with a primary myelofibrosis.

## Conclusions

The simultaneous occurrence of multiple myeloma and JAK2 positive myeloproliferative neoplasms is possible, although the underlying mechanism is not very well understood. For an accurate diagnosis, the use of molecular methods and the elimination of any confusing situations such as fibrosis associated to multiple myeloma, and, respectively, pancytosis via splenic amyloidosis associated to multiple myeloma, are recommended.

**Acknowledgement:** part of this study (implementation of Free Light Chain assay and molecular biology method for JAK2 identification) was funded by grant CEEX 74/ 2006 and PN 41-087/ 2007 from the Romanian Ministry of Research and Technology.
